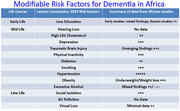# Risk Factors and Genetics of Dementia in Africa

**DOI:** 10.1002/alz70860_098464

**Published:** 2025-12-23

**Authors:** Rufus O. Akinyemi

**Affiliations:** ^1^ Institute of Advanced Medical Research and Training, College of Medicine, University of Ibadan, Ibadan, Oyo State, Nigeria

## Abstract

**Background:**

The burden of dementia in Africa is projected to grow exponentially by the year 2050 driven by population growth, population ageing and other factors. There is an heterogenous body of knowledge on modifiable risk factors but limited understanding of the genetic architecture of dementia in Africa.

**Methods:**

In the “Recruitment and Retention for Alzheimer's Disease Diversity Genetic Cohorts in the ADSP (READD‐ADSP)” genome‐wide genotyping was performed on 91 AD cases and 97 cognitive unimpaired controls from Nigeria and Ghana. APOE alleles and ABCA7 deletion (rs142076058) were sequenced using Sanger. Systematic review of the non‐genetic risk factors for dementia in Africa was undertaken.

**Results:**

Using the framework of the Lancet Commission 2024 iteration, the 14 modifiable risk factors have been reported in Africa with the exception of air pollution and hearing loss and sparse data on visual loss. Overall hypertension, diabetes, dyslipidemia, low education, social isolation and low socio‐economic status are prominent risk factors while infections like HIV/AIDS, malaria, toxoplasmosis and onchocerciasis have also been associated. There is an urgent need for large datasets on risk factors for dementia in Africa that will enable accurate computation of population attributable fraction (PAF) for the key risk factors.

Research on the genetics of dementia in Africa is in infancy, but existing data from candidate gene studies demonstrate variation in the contribution of APO4 e4 allele to AD with significant association in northern Africa but attenuation in SSA. Among the genetic loci examined, several showed nominal significance. Notably, the most prominent marker was found in SORL1 (rs17125523; *p* = 2x10‐3). Additionally, we discovered an exonic nonsynonymous marker in the BIN1 gene (rs112318500), which is specific to African ancestry and showed a protective effect. APOE e4 allele showed a significant association with AD risk (OR=2.5; CI:1.5‐4.2; pv=0.001), while the e2 indicated a protective trend but did not reach statistical significance. No statistical difference in the frequency of ABCA7 deletion was observed between AD and CU individuals.

**Conclusion:**

Our preliminary findings potentially offering new insights into the genetic underpinnings of AD. Data collection is ongoing across multiple sites in Africa.